# Supplementary material for: Content-rich biological network constructed by mining PubMed abstracts
Source: BMC Bioinformatics. 2004 Oct 8;5:147. doi: 10.1186/1471-2105-5-147 (PMC528731; doi:10.1186/1471-2105-5-147)
Supplement: Additional File 2 — The original results of the above study (non-essential files are deleted to keep the file size under the limit set by BMC bioinformatics). [file 1471-2105-5-147-S2.bz2 › chilibotAdditionalFile2/dip05/692ID12004122E18014/html/BTUC_BTUD.html]

 


 **BTUC** and **BTUD** 
  
Found 7 abstracts in PubMed, retrieved 05.  
 

 What does Google say? 
 PDF only 
| .edu only 

---

**Interactive relationship** (e.g. stimulation, inhibition, etc)

**Neutral relationship**- The two ATP binding cassettes  **BtuD**  are in close contact with each other, as are the two membrane spanning subunits  **BtuC** .  Ref: 12004122 Science, 2002

**Non-interactive relationship** (e.g. studied together, co-existance, homology, etc.)

- In addition to this plasmid with a 6 kilobase insert of S. typhimurium DNA, vitamin B12 utilization by E. coli btuB strains required the  **btuC**  and  **btuD**  products, necessary for transport across the cytoplasmic membrane, but not the btuE or tonB product.  Ref: 2656634 J Bacteriol, 1989
- The  **BtuC**  subunits provide 20 transmembrane helices grouped around a translocation pathway that is closed to the cytoplasm by a gate region whereas the dimer arrangement of the  **BtuD**  subunits resembles the ATP bound form of the Rad50 DNA repair enzyme.  Ref: 12004122 Science, 2002
- Hence, null mutations in  **btuC**  and  **btuD**  were constructed and revealed that the  **btuC**  mutant had a strong impairment similar to that of the btuF mutant.  Ref: 11790740 J Bacteriol, 2002
- The growth response to CN Cbl of the btuF mutant was much stronger than the slight impairment previously described for  **btuC** ,  **btuD** , or btuF mutants.  Ref: 11790740 J Bacteriol, 2002
- Transport of vitamin B12 across the cytoplasmic membrane of Escherichia coli requires the products of  **btuC**  and  **btuD** , two genes in the btuCED operon.  Ref: 2671656 Mol Gen Genet, 1989
- Transport across the cytoplasmic membrane CM requires the  **BtuC**  and  **BtuD**  proteins, which are most related in sequence to the transmembrane and ATP binding cassette proteins of periplasmic permeases for iron siderophore transport.  Ref: 11790740 J Bacteriol, 2002
